# Scenario-Based Serious Game for Screening Mild Cognitive Impairment in Older Adults: Cross-Sectional Preliminary Validation Study

**DOI:** 10.2196/92334

**Published:** 2026-09-16

**Authors:** Bomyi Jeon, Chi Hyeon Noh, Soo Rim Noh, Yerin Shim, Seung Bin Cho, Sungkun Cho

**Affiliations:** 1 Department of Psychology, Chungnam National University, 99 Daehak-ro, Yuseong-gu, Daejeon, 34134, Republic of Korea, 82 42-821-6366; 2 Department of Psychology, Pusan National University, Pusan, Republic of Korea

**Keywords:** mild cognitive impairment, serious games, digital health, cognitive screening, machine learning, diagnostic accuracy, ecological validity, dementia

## Abstract

**Background:**

Many existing digital cognitive assessments rely on isolated or abstract tasks, and primarily use accuracy-based outcome measures, despite recent advances in the field. Few have been culturally adapted to reflect the familiar daily-life experiences of Korean older adults. Integrating culturally meaningful scenarios with task-specific accuracy and reaction time (RT) measures may provide a more ecologically grounded approach to mild cognitive impairment (MCI) screening.

**Objective:**

This study aimed to develop and preliminarily evaluate a tablet-based, scenario-based serious game for community-based MCI screening among Korean older adults.

**Methods:**

In this cross-sectional diagnostic accuracy study, community-dwelling adults aged 60‐84 years were recruited through convenience sampling from a senior welfare center in Daejeon, South Korea, between July 2024 and June 2025. Of 87 participants who underwent eligibility assessment, 67 participants were included in the analysis: 47 cognitively normal participants, and 20 participants with MCI. Clinical classification was determined according to Petersen criteria based on the Korean version of the Consortium to Establish a Registry for Alzheimer’s Disease battery, second edition, structured clinical interviews, and clinician judgment blinded to the serious game results. The serious game initially comprised 5 culturally familiar and ecologically grounded daily-life tasks, from which task-specific accuracy and RT features were extracted. A reduced 4-task model informed by exploratory misclassification analysis served as the primary classification model and was evaluated using random forest classification with out-of-bag validation. A full 5-task model and a demographic-adjusted model were also evaluated. Convergent validity and perceived workload were assessed.

**Results:**

The group with MCI was older (77.3 vs 74.5 y; *P*=.002) and had fewer years of education (9.4 vs 11.2 y; *P*=.04). The primary 4-task model achieved an overall accuracy of 82.1% (55/67), balanced accuracy of 80.1%, sensitivity of 75% (15/20), specificity of 85.1% (40/47), and an area under the receiver operating characteristic curve of 0.787 (95% CI 0.643‐0.932). The demographic-adjusted model showed broadly comparable discrimination (area under the receiver operating characteristic curve 0.794, 95% CI 0.654‐0.934). Accuracy and RT measures from the sale items task ranked among the most important predictors. Digital composite scores were significantly correlated with the Korean version of the Consortium to Establish a Registry for Alzheimer’s Disease battery, second edition total score (*r*=.655 and *r*=.447; both *P*<.001). Perceived workload did not significantly differ between groups (*P*=.97).

**Conclusions:**

This study provides preliminary evidence for the diagnostic utility and feasibility of a tablet-based serious game integrating culturally familiar daily-life scenarios with task-specific accuracy and RT measures, thereby capturing both performance accuracy and potential differences in processing efficiency. The automated format may offer a scalable and accessible screening option for community settings without specialized equipment. Independent, multisite, and longitudinal validation is required to establish its generalizability and predictive utility.

## Introduction

Mild cognitive impairment (MCI) represents an intermediate clinical state between normative aging and dementia, characterized by cognitive decline that does not yet interfere substantially with daily functioning [1-3]. Individuals with MCI show an elevated risk of progressing to various dementia syndromes, although clinical trajectories remain heterogeneous [4-6]. The growing global burden of dementia, which is projected to exceed 130 million cases by 2050, highlights the importance of identifying cognitive vulnerability as early as possible [7]. Early detection enables timely monitoring, preventive intervention, and implementation of lifestyle strategies that may mitigate further decline [8,9]. In South Korea, nationwide prevalence estimates indicate that 20% to 30% of older adults meet criteria for MCI, underscoring the need for scalable screening approaches [10]. However, current diagnostic standards are often constrained by lengthy administration times, high costs, and the need for specialized personnel [11,12]. Furthermore, because early cognitive changes often mimic normal age-related decline [13], many individuals lack insight into their clinical status and fail to seek medical attention voluntarily [14]. This inherent difficulty in self-recognition, coupled with systemic barriers, underscores the need for accessible, cost-effective, and automated community-level screening tools.

Traditional neuropsychological assessments used for MCI screening vary widely in diagnostic performance [15,16]. The Mini-Mental State Examination (MMSE), although widely administered, shows low sensitivity for detecting MCI due to ceiling effects and limited assessment of attention and executive functioning [15,17,18]. The MoCA (Montreal Cognitive Assessment) demonstrates higher sensitivity (often 80%‐100%) but tends to show lower specificity and still requires trained examiners, which limits its scalability for routine community-based screening [15,17,19]. In South Korea, the Korean version of the Consortium to Establish a Registry for Alzheimer’s Disease is widely used as a clinical reference battery because it provides well-established normative data within an internationally standardized assessment framework [20,21]. Its strong emphasis on memory assessment, reflecting its original development for Alzheimer disease, may reduce its sensitivity to nonamnestic presentations of MCI [22,23]. These limitations provide a rationale for digital approaches that can complement conventional screening. However, advances in digital approaches alone do not ensure that these tools are culturally appropriate or sufficiently sensitive to detect subtle behavioral changes associated with early cognitive decline.

Digital cognitive assessment has advanced substantially in recent years, with remote, unsupervised, and tablet-based tools demonstrating promising diagnostic performance for detecting MCI and dementia [24-27]. Serious game–based approaches have further extended digital assessment by embedding cognitive tasks within familiar everyday activities [28-31]. For example, recent scenario-based tools have assessed cognitive functioning through activities such as shopping in a virtual supermarket and other self-administered tasks modeled on everyday situations, supporting the feasibility of ecologically valid MCI screening [32-34]. However, these tools were developed and validated outside South Korea, and their language, scenarios, and interaction demands may not transfer directly to community-dwelling Korean older adults. Consistent with this, reviews of touchscreen cognitive tools have identified limited cultural and linguistic diversity in their development and validation, raising concerns about the generalizability of these tools across populations [35]. Culturally adapted assessment therefore requires more than translating existing tools; it involves designing task content, instructions, and interaction formats to reflect the everyday experiences of the intended population [36,37]. These limitations highlight the need for culturally adapted, scenario-based digital assessments developed and validated specifically for Korean older adults.

Culturally adapted design addresses one limitation of existing digital cognitive assessments; however, another limitation is their continued reliance on summary end point measures, such as overall accuracy, total completion time, or other aggregate scores [38,39]. Although these summary indices provide useful information regarding overall task performance, they may not fully capture subtle cognitive changes associated with early-stage MCI [40]. In contrast, a process-based approach to cognitive assessment offers an alternative framework by focusing on how cognitive tasks are performed rather than solely on their outcomes [38,39]. Within this framework, behavioral patterns observed during task performance provide clinically meaningful information beyond conventional end point scores [38,39]. Digital cognitive assessment is particularly well suited to process-based evaluation because it enables the automated capture of response-process data [41]. Such process-sensitive measures include reaction time (RT) and other behavioral indicators derived from task performance that are difficult to obtain using conventional paper-and-pencil assessments [41]. This perspective is consistent with information-processing and processing-speed theories of cognitive aging, which suggest that reduced processing efficiency associated with aging and early cognitive decline may be reflected in behavioral measures such as RT [42,43]. Among these process-sensitive behavioral indicators, the present study focused specifically on task-level RT, which may reflect processing efficiency while providing information complementary to conventional accuracy-based scores [44-48].

To address these remaining gaps, we developed a tablet-based, scenario-based serious game designed from the outset around the cultural and everyday experiences of community-dwelling Korean older adults. Drawing on a process-based assessment framework, the platform integrates culturally familiar daily-life scenarios with task-level accuracy and RT measures, a process-sensitive indicator of cognitive performance [38,41]. Task-level RT was included as a potential indicator of processing efficiency, complementing conventional accuracy-focused assessment [38,39]. This cross-sectional diagnostic accuracy study evaluated the preliminary diagnostic performance, convergent validity, and feasibility of this integrated approach. We further examined the contribution of task-level accuracy and RT measures to classifying older adults as having MCI or normal cognition.

## Methods

### Participants

#### Inclusion and Exclusion Criteria

Eligibility criteria included community-dwelling older adults aged 60‐84 years who voluntarily participated and provided informed consent.

Exclusion criteria were the following: (1) a diagnosis of major neurocognitive disorder or inability to perform activities of daily living independently; (2) a history of major psychotic disorders (eg, schizophrenia) or current use of antipsychotic medications; (3) a history of significant head injury or neurological disorders; (4) uncorrectable hearing impairment or physical conditions that precluded tablet use; or (5) difficulty understanding the study aims and procedures. Additionally, participants scoring ≤19 on the MMSE, indicative of at least moderate neurocognitive impairment [49], were excluded to ensure that the sample targeted the MCI and cognitively normal (CN) range. However, no enrolled participant met this exclusion criterion.

#### Participants’ Characteristics

This cross-sectional diagnostic accuracy study included community-dwelling adults aged 60‐84 years from Daejeon, South Korea.

#### Sampling and Recruitment Procedure

Participants were recruited using convenience sampling from the Yuseong Senior Welfare Center in Daejeon, South Korea. The research team visited the center, explained this study to potential participants, and invited voluntary participation, resulting in a self-selected sample. A total of 89 individuals expressed interest in this study, of whom 2 individuals voluntarily withdrew during the scheduling process before formal screening. Consequently, 87 participants underwent eligibility assessment. Data collection was conducted from July 2024 to June 2025 in a quiet, partitioned consultation room within the facility to minimize environmental distractions and participant fatigue.

### Ethical Considerations

This study was approved by the Institutional Review Board of Chungnam National University (202403-SB-038-01) and conducted in accordance with the Declaration of Helsinki. All participants received a detailed explanation of this study’s purpose, procedures, potential risks, and their right to withdraw at any time without penalty, and provided written informed consent before participation. To protect privacy and confidentiality, all data were deidentified using unique identification numbers, and electronic files were stored in password-protected folders accessible only to the research team. Participants received a clinical report on their cognitive status and a participation fee of 50,000 KRW (a currency exchange rate of 1000 KRW=US $0.74 was applicable; approximately US $37) via bank transfer for the 2-hour session. The compensation was determined to be appropriate for the time commitment and did not constitute undue inducement. No identifiable personal information is included in this paper.

### Sample Size, Power, and Precision

No a priori power analysis was conducted as this was a preliminary validation study focusing on feasibility and estimation precision rather than hypothesis testing. The target sample size was pragmatically set at approximately 60‐70 participants based on recruitment feasibility and consistency with prior early-stage pilot and validation studies, which typically enroll small-to-moderate samples [50]. A total of 67 participants were included in the final analysis. To address precision, 95% CIs are reported for all primary diagnostic metrics, and findings are interpreted based on the precision of these estimates rather than reliance on *P* values alone. These considerations are consistent with recommendations for pilot and preliminary diagnostic accuracy studies [51,52].

### Measures and Covariates

#### Overview

The primary measures in this study were performance indices derived from the serious game, which were used as features for diagnostic classification. Secondary measures included the second edition of the Korean version of the Consortium to Establish a Registry for Alzheimer’s Disease (CERAD-K2) total score, which was calculated from selected core subtests according to the standardized scoring protocol and used exclusively for convergent validity analysis. The NASA Task Load Index (NASA-TLX) was used to assess perceived workload and usability of the digital tool. Age and education were included as covariates because baseline group differences were observed in these variables, and they were considered potential confounders in the interpretation of diagnostic performance.

#### Reference Standard

The CERAD-K2 battery was used as part of the clinical reference standard [53]. During the clinical interview, a brief *DSM-5* (*Diagnostic and Statistical Manual of Mental Disorders* [Fifth Edition])–based prompt was used to document participants’ self-reported cognitive difficulties across domains as supplementary clinical information. The final diagnosis of MCI vs CN was determined by a clinician based on a comprehensive assessment, including all CERAD-K2 subtest results and structured clinical rating procedures, with consideration of potential confounding factors (eg, affective disorders). Additional measures, including the MMSE (J3) and executive function tests (eg, Trail Making Test and Stroop Test; J9, J-ga), were used to inform clinical diagnosis, although they were not included in the CERAD-K2 total score. The clinician was blinded to the results of the serious game assessment during diagnostic determination.

#### Convergent Validity Measure

Separately, the CERAD-K2 total score (maximum score of 110) was derived from the sum of its 7 core components (J1, J2, and J4-J8) solely for the purpose of assessing the convergent validity of the digital tool. Following the standardized CERAD-K2 scoring protocol, J3 (MMSE) and executive function measures (Trail Making Test and Stroop; eg, J9 and J-ga) were excluded from the total score.

#### Serious Game Tasks

The serious-game screening tool comprised 5 ecologically valid tasks that probe early vulnerable neurocognitive functions in older adults. Accuracy and RT were recorded for each task to index both performance level and processing efficiency. Tasks were embedded in a continuous daily-life scenario that approximates instrumental activities of daily living in older adults. Their scenario roles and targeted cognitive domains are summarized in Table 1. Representative screenshots of the user interface for each task are provided in Figures S1-S5 in Multimedia Appendix 1. All 5 tasks were administered, and features from 4 tasks were ultimately retained for the machine learning (ML) classification model (see Results section for details on model refinement).

Each task was preceded by 1 practice trial to ensure rule comprehension. Accuracy was calculated as the raw number of correct responses (or partial score where applicable), and RT was measured in milliseconds.

Regarding sale items (memory), participants were presented with a list of 12 target items to memorize. The items consisted of high-frequency bisyllabic Korean words representing common market goods. In the recognition phase, participants had to identify the 12 target items from a pool containing 12 distractors (24 items in total). The performance metrics included accuracy (number of correctly identified items) and total completion time (from task onset to completion).

Regarding puzzle-solving (visuospatial), participants were asked to complete a puzzle consisting of 9 pieces. The metrics recorded were accuracy (success or failure) and total completion time (mean time from start to placing the last piece).

Regarding traffic signals (executive function and set-shifting), this task assessed cognitive flexibility using 20 trials with a 2-second time limit per trial. Stimuli consisted of 4 variations: 2 colors (green and red) and 2 postures (walking and standing). The task required participants to respond (“go” or “stop”) based on a changing rule (color rule vs shape rule). For example, under the “color rule,” participants had to follow the color regardless of the shape, whereas under the “shape rule,” they had to attend to the posture. Metrics included accuracy (number of correct responses) and mean RT (average latency of correct responses).

Regarding rice-barley (attention and inhibition)*,* a localized auditory go or no-go task consisting of 100 trials was administered. Participants heard randomized voice stimuli of “rice” (ssal; go) or “barley” (bori; no-go) with a 1-second time limit. The ratio of go to no-go stimuli was 7:3 (70 rice and 30 barley) to induce response prepotency. Metrics included accuracy (number of correct responses) and mean RT (average latency for correct “go” trials).

Regarding spatial navigation (visuospatial memory), participants viewed a map with a designated path and were required to reproduce the path by connecting dots. The task consisted of 3 trials. While the practice trial presented the map in a standard orientation, the test trials required mental rotation, as the maps were rotated 90°, 180°, and 270°. Metrics included accuracy (partial scoring based on the number of correctly connected segments) and mean completion time (average time to complete the 3 trials).

**Table 1.** Task components of the serious game–based mild cognitive impairment screening tool. The tool consists of 5 ecologically grounded tasks designed to approximate instrumental activities of daily living and assess early neurocognitive changes. These tasks were used in a cross-sectional diagnostic study involving community-dwelling older adults (N=67) at the Yuseong Senior Welfare Center in Daejeon, South Korea, between July 2024 and June 2025. Scenario phases are numbered according to their sequence within the continuous daily-life storyline. Accuracy and reaction time were recorded for each task to assess performance level and processing efficiency. Five tasks were embedded in a continuous daily-life scenario approximating instrumental activities of daily living in older adults. Each task was preceded by 1 practice trial.

Scenario phase	Task name	Scenario role	Targeted domain
1 and 5	Sale items task	Checking a market flyer to encode discounted items (step 1) and later recognizing and purchasing those items at the market (step 5)	Multimodal (visual-verbal) memory
2	Puzzle-solving	Helping a grandchild complete a puzzle	Visual organization
3	Traffic signal	Crossing a broken traffic light on the way to the market	Executive function (interference control, set-shifting)
4	Rice-barley go/no-go	Participating in a local promotion (rice-barley; a Korean traditional hand game) at the market	Complex attention (sustained and selective attention, inhibitory control)
6	Spatial navigation	Finding the way back home	Spatio-temporal memory, route learning

#### Demographic Covariates

Age and education were included as demographic covariates because statistically significant group differences were observed in these variables. These variables were not prespecified predictors but were included to account for potential confounding in the interpretation of diagnostic performance. This approach is consistent with the common practice in diagnostic accuracy studies to adjust for demographic factors associated with cognitive performance [54,55].

#### Usability Assessment and Psychometric Evaluation

Usability was evaluated immediately after the digital protocol using the NASA-TLX [56], a self-report instrument widely used to measure subjective mental workload. The assessment used items based on the 6 dimensions of NASA-TLX (mental demand, physical demand, temporal demand, performance, effort, and frustration) to evaluate the perceived workload and usability of the digital tool.

### Procedure

#### Data Collection

All sessions were conducted individually in a quiet room. Following informed consent, participants first completed the MMSE for initial screening, the serious game assessment (approximately 20 min), and the usability questionnaire. After a 10-minute rest, the CERAD-K2 battery and clinical interviews were administered using paper-and-pencil procedures, with optional breaks provided to minimize fatigue.

#### Quality of Measurements

Assessments were administered by 2 master’s-level trainees who received prior training in the standardized assessment procedures. All sessions were conducted under the supervision of a licensed clinical psychologist, who reviewed the assessment results and ensured consistency in administration and diagnostic decision-making.

#### Clinical Classification

Participants were classified into CN groups or groups with MCI according to Petersen’s criteria. Objective cognitive impairment was defined as performance at least 1.5 SD below the age- and education-adjusted norms (≤−1.5 SD) in one or more cognitive domains. Subjective cognitive complaints were assessed during the clinical interview. Clinical diagnosis was based on a comprehensive assessment that included the CERAD-K2 neuropsychological battery and a structured clinical interview. All neuropsychological assessments and interviews were administered by 2 master’s-level clinical psychology trainees. Final diagnoses were determined by a licensed clinical psychologist who supervised the process and reviewed all test and interview data.

Participants in the group with MCI did not exhibit significant impairment in activities of daily living. In addition to the prespecified exclusion criteria, a subset of participants was excluded after the clinical interview based on semistructured evaluation. Participants were excluded if the interview suggested that their cognitive complaints were primarily attributable to current depressive or anxiety symptoms (suspected affective etiology) or if functional impairment in activities of daily living exceeded the range typically associated with MCI, indicating possible major neurocognitive disorder. These decisions were made by a licensed clinical psychologist based on clinical judgment. This approach was intended to reduce potential confounding related to affective symptoms or functional impairment.

### Data Diagnostics

#### Data Preprocessing

Before model construction, we preprocessed the data to ensure quality. Missing values resulting from technical errors or system malfunctions were excluded via listwise deletion; the number of affected observations ranged from 1 to 7 across behavioral features.

The wide range of RT values across features (approximately 270 to 296,000 ms) reflects differences in how RT was operationalized across tasks, not extreme values within any single task. The traffic signal and rice-barley RT represent mean per-trial response latencies, whereas sale items and spatial navigation RT represent total task completion times. All recorded RT values were retained, as no system errors were flagged during data logging.

All behavioral features (accuracy and RT for each of the 4 tasks; 8 features total) and demographic covariates (age and year of education) were then standardized to *z* scores (mean 0, SD 1) across the full analytic sample using the scale() function in R (R Foundation) because random forest (RF) splits are based on the rank order of observations; tree-based classifiers are invariant to monotonic transformations of individual predictors [57,58]; standardization does not alter the model’s splitting behavior. We applied standardization for 2 practical reasons. First, the scale differences described above make unstandardized feature values difficult to compare in descriptive summaries and variable importance plots. Second, the digital composite scores (DCS1 and DCS2) are computed as Gini-weighted sums of feature values, and without standardization, features on larger absolute scales would dominate these composites. As standardization parameters (sample mean and SD) were computed from the full analytic sample before out-of-bag evaluation, this procedure is not strictly leakage-free. In practice, however, *z* scoring preserves the rank order on which RF splits depend, so out-of-bag predictions remain unaffected [58]. The *m*_*try*_ parameter was set to 3—the square root of the number of behavioral predictors—following the standard recommendation for classification [57].

#### ML Classification

We used the RF algorithm to classify individuals with MCI and CN. RF is an ensemble ML algorithm known to be particularly effective for datasets characterized by small sample sizes and class imbalance [57,59]. The CN-to-MCI ratio in our sample reflects the natural prevalence of MCI in older general populations, where early cognitive decline often goes undetected because of preserved activities of daily living [10]. Unlike traditional parametric models such as logistic regression, RF handles high-dimensional feature spaces and nonlinear interactions without making strict distributional assumptions while maintaining robustness against overfitting through its recursive partitioning and bagging mechanisms [59,60].

The RF model was optimized with 500 decision trees ($n_{tree}\text{=}500$). To ensure optimal variance reduction and model stability, the number of features randomly sampled at each node split ($m_{try}$) was set to 3, which corresponds to the square root of the total number of behavioral predictors, a recommended standard heuristic for classification tasks [60].

Model performance and generalization error were strictly evaluated using the out-of-bag procedure, which serves as a rigorous internal validation mechanism [60]. Each tree of RF used 63.2% of the original data as a bootstrap sample for training and set the remaining 36.8% of observations as an out-of-bag sample, which was excluded from the training of that specific tree. These out-of-bag samples then functioned as an independent validation set for each tree. By aggregating classification votes across the entire forest for each observation only when it was in an out-of-bag state, the model provides an unbiased estimate of generalization performance (eg, accuracy, sensitivity, specificity, and area under the receiver operating characteristic curve [AUC]). This approach effectively mitigates the risk of data leakage and provides a reliable assessment of diagnostic accuracy in studies where a separate hold-out test set is not feasible due to sample size constraints [60,61]. All statistical analyses were implemented using the *randomForest* package [61] in R (version 4.3.0) [62].

#### DCS

The primary outputs of the diagnostic model were DCS1 and DCS2, constructed to separately examine the contribution of accuracy-based performance and time-based processing efficiency. For both composites, feature weights were derived from a preliminary RF model trained on the present sample to maximize the contribution of task variables that best discriminated MCI from CN. DCS1 (accuracy-weighted) was calculated as a weighted sum of accuracy scores only, providing a composite analogous to traditional neuropsychological indices. In contrast, DCS2 (RT-included) incorporated both accuracy and RT features in a weighted sum to capture potential slowing and efficiency changes that may emerge in early cognitive decline even when accuracy remains relatively preserved.

#### Classification Performance and Convergent Validity

Model performance was assessed using accuracy, sensitivity, specificity, balanced accuracy, and the AUC. We estimated the 95% CI for the AUC using the DeLong method [63], implemented via the ci.auc() function in the *pROC* package in R; this approach derives the variance of the AUC nonparametrically from each observation’s structural contribution to the overall estimate without assuming a specific score distribution. Feature importance values were extracted using the mean decrease in Gini impurity to examine the relative contribution of each task variable. Convergent validity was assessed by calculating the Pearson correlation coefficient between the digital tool performance and the final CERAD-K2 total score, with 95% CIs estimated using Fisher z transformation.

## Results

### Participant Flow

**Figure 1.**
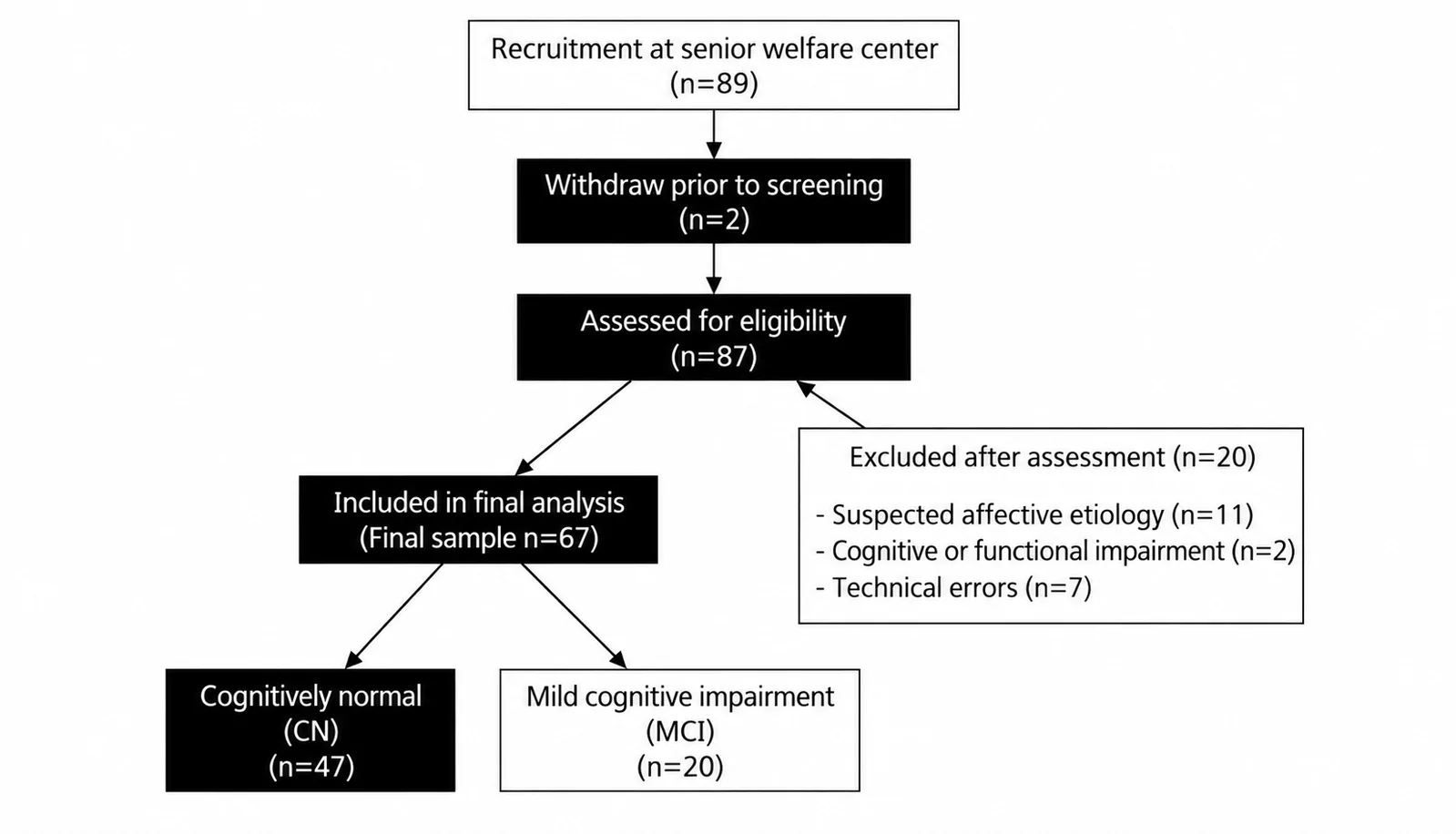
Participant flow diagram illustrating recruitment, screening, and final inclusion. CN: cognitively normal; MCI: mild cognitive impairment.

Recruitment and data collection were conducted from July 2024 to June 2025. A total of 89 individuals expressed interest in this study, of whom 2 withdrew before formal screening. Consequently, 87 participants underwent eligibility assessment. No participants were excluded at the screening stage based on the prespecified criteria. Following data collection, 20 participants were excluded from the final analysis due to suspected affective etiology (n=11), impairment exceeding the MCI range (n=2), or technical errors in data logging (n=7). This resulted in a final analytic sample of 67 (47 CN and 20 MCI) participants. A detailed participant flow diagram, including screening and exclusion reasons, is presented in Figure 1.

The diagram shows the recruitment, screening, withdrawal, exclusion, and final inclusion of community-dwelling older adults aged 60‐84 years in Daejeon, South Korea, resulting in a final analytic sample of 67 (47 CN and 20 MCI) participants.

### Clinical Characteristics and Group Validation

The final analytic sample included 67 older adults, comprising 47 CN participants and 20 participants with MCI. Demographic and lifestyle characteristics of the groups are summarized in Table 2. Independent-samples *t* tests were used for continuous variables, and chi-square tests were used for categorical variables to compare baseline characteristics between groups.

Statistically significant differences were observed in age (*P*=.002) and education (*P*=.04). The group with MCI (77, SD 3 y) was significantly older and had fewer years of education (9, SD 4 y) than the CN group (74, SD 5, and 11, SD 2 y, respectively).

No significant differences were observed between groups in other demographic variables. The proportions of females were similar between groups (32/47, 68%, vs 14/20, 70%; *P*=.99), and no statistically significant differences were found in marital status (*P*=.65). However, no statistically significant difference was observed in driving status (*P*=.051), although a lower proportion of participants with MCI reported driving (6/20, 30%) compared with CN participants (28/47, 60%).

To characterize the clinical profiles of the groups and examine the validity of the classification, CERAD-K2 total scores were compared between groups. As expected, the CN group scored significantly higher than the group with MCI (*P*<.001; Table 2). This difference is consistent with the expected clinical distinction between the groups and supports the diagnostic classification based on the clinical reference standard.

**Table 2.** Participant characteristics and CERAD-K2^a^ total score by group. This cross-sectional diagnostic study compared CN^b^ older adults and those with MCI^c^. Participants were recruited from the Yuseong Senior Welfare Center in Daejeon, South Korea, between July 2024 and June 2025 (total N=67; 47 CN and 20 MCI). Independent samples *t* tests were used for continuous variables, and chi-square tests were used for categorical variables.

Variable	CN (n=47)	MCI (n=20)	*P* value
Demographics			
Age (y), mean (SD)	74 (5)	77 (3)	.002
Education (y), mean (SD)	11 (2)	9 (4)	.04
Female, n (%)	32 (68)	14 (70)	.99
Married, n (%)	43 (91)	18 (90)	.65
Currently driving, n (%)	28 (60)	6 (30)	.051
Clinical measures			
CERAD-K2 total score, mean (SD)	81.0 (9.0)	64.0 (8.0)	<.001

a CERAD-K2: Consortium to Establish a Registry for Alzheimer's Disease Assessment Packet, Korea, second edition.

b CN: cognitively normal.

c MCI: mild cognitive impairment.

### Classification Performance, Sensitivity Analysis, and Feature Importance

As a baseline analysis, we evaluated the full 5-task battery, including the puzzle-solving task. In this initial model, classification performance was 79.1% (53/67) accuracy, 70% (14/20) sensitivity, 83% (39/47) specificity, and an AUC of 0.861 (95% CI 0.757‐0.964). These findings provide a reference for the diagnostic performance of the complete scenario-based assessment (Table 3).

**Table 3.** Confusion matrix for the full five-task model. This cross-sectional diagnostic study evaluated the classification performance of 3 models in distinguishing between CN^a^ older adults and those with MCI^b^. Participants were recruited from the Yuseong Senior Welfare Center in Daejeon, South Korea, between July 2024 and June 2025 (N=67). Out-of-bag predictions for the full five-task model are presented here (see Tables 4 and 5 for the primary four-task and demographic-adjusted models, respectively).

	Observed CN, n	Observed MCI, n
Predicted CN	39	6
Predicted MCI	8	14

a CN: cognitively normal.

b MCI: mild cognitive impairment.

To explore potential sources of misclassification, we conducted an exploratory analysis of task-level performance patterns. This analysis indicated that the puzzle-solving task was associated with a higher proportion of false-negative classifications, with some participants with MCI showing relatively preserved performance on this task. Specifically, participants with MCI misclassified as CN showed higher accuracy (*P*=.06) and faster completion times (*P*=.01) than those correctly identified as MCI.

Based on these observations, we conducted a sensitivity analysis excluding the puzzle-solving task. In this reduced model, sensitivity increased to 75% (15/20), while overall accuracy (55/67, 82.1%) and specificity (40/47, 85.1%) remained comparable. The AUC decreased to 0.787 (95% CI 0.643‐0.932). The final RF model correctly identified 15 of 20 cases with MCI and 40 of 47 CN cases, as detailed in Table 4.

**Table 4.** Confusion matrix for the primary 4-task model. This cross-sectional diagnostic study involved community-dwelling older adults (N=67) recruited from the Yuseong Senior Welfare Center in Daejeon, South Korea, between July 2024 and June 2025.

	Observed CN^a^, n	Observed MCI^b^, n
Predicted CN	40	5
Predicted MCI	7	15

a CN: cognitively normal.

b MCI: mild cognitive impairment.

**Figure 2.**
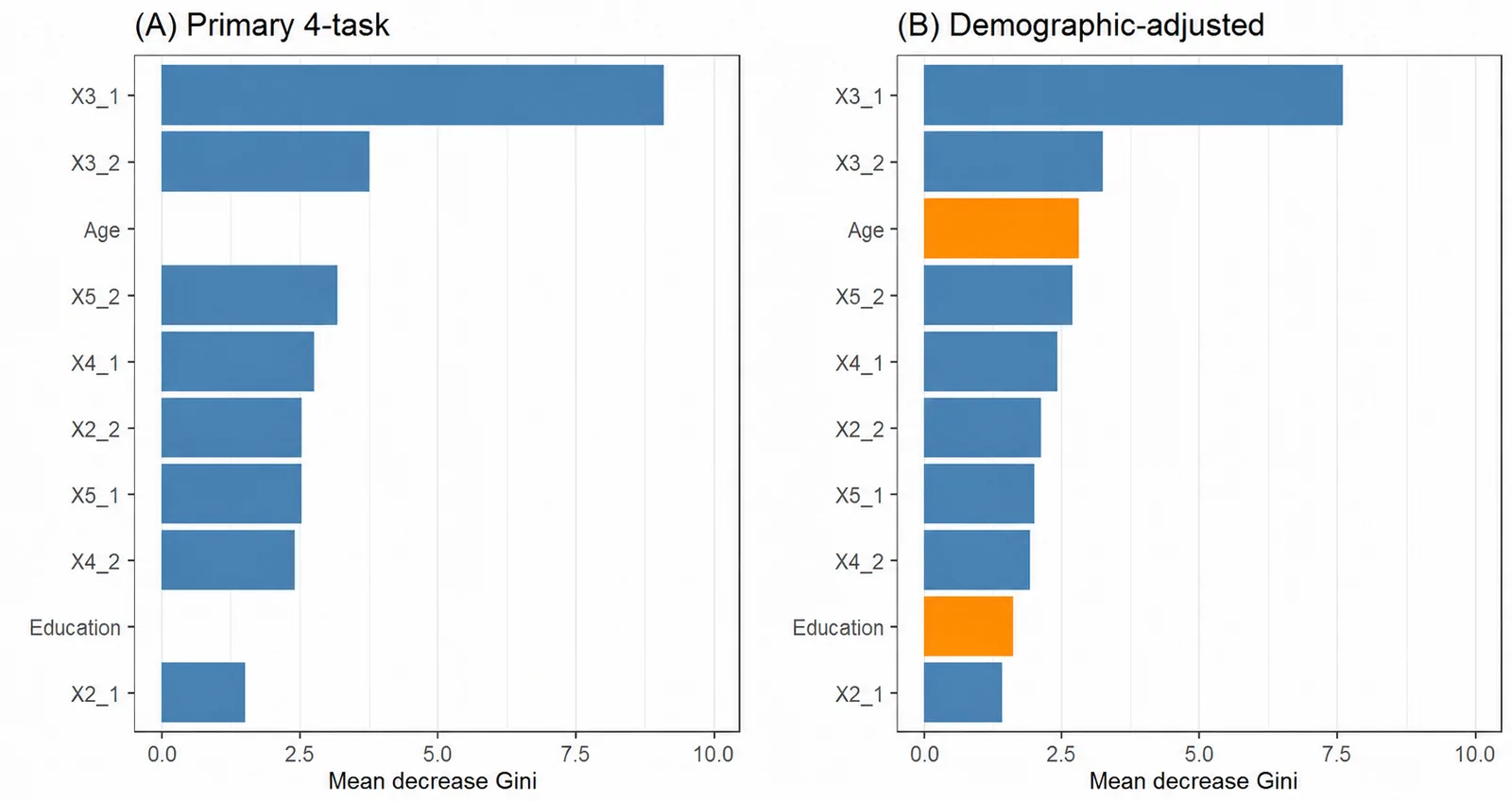
Variable importance comparison between primary 4-task and demographic-adjusted models. Variable importance was ranked based on the mean decrease in Gini impurity. (A) Primary 4-task model using 8 digital behavioral features. (B) Demographic-adjusted model including age and education. Blue bars indicate behavioral features, whereas orange bars represent demographic covariates. The sale items total score (X3_1) was the highest-ranked feature across both models. This figure is based on a cross-sectional diagnostic study involving community-dwelling older adults recruited from the Yuseong Senior Welfare Center in Daejeon, South Korea, between July 2024 and June 2025.

The analysis of feature importance, based on the mean decrease in Gini impurity, identified the variables that contributed most to the model’s classification performance (Figures 2A and 2B). The most important feature for distinguishing MCI from CN was the sale items total score (X3_1), which reflects visual-verbal memory and decision-making. Following this, RT-related variables also contributed substantially to classification. The next most important features included sale items RT (X3_2), traffic signal overall RT (X2_2), and spatial navigation RT (X5_2). The prominence of RT-related features, particularly those associated with executive function and processing speed, suggests that variability in response speed may be relevant for distinguishing early cognitive decline.

### Assessment of Demographic Covariates on Model Robustness

**Figure 3.**
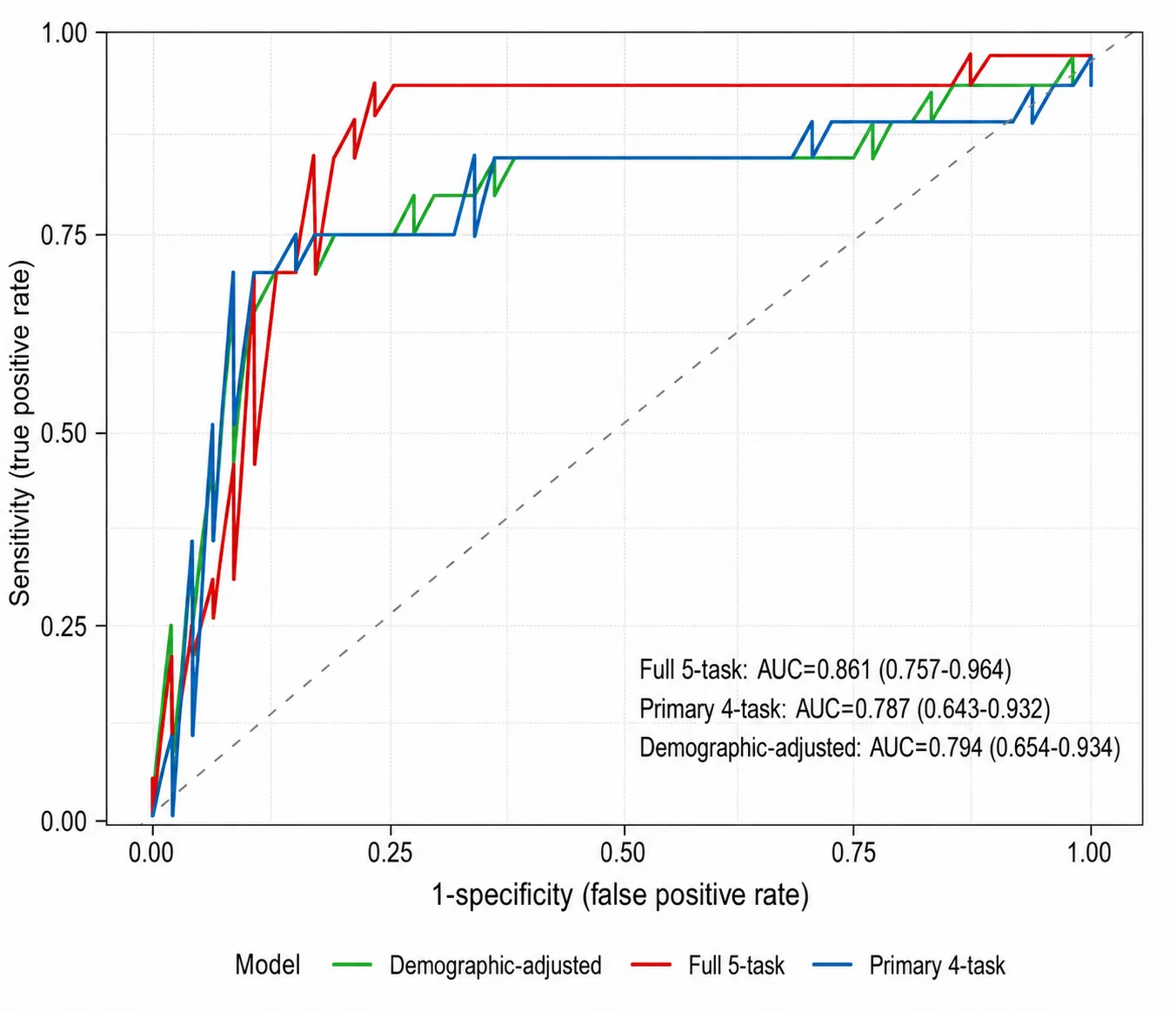
ROC curve comparison of the 3 predictive models for distinguishing between CN and MCI. This cross-sectional diagnostic study involved community-dwelling older adults (N=67) recruited from the Yuseong Senior Welfare Center in Daejeon, South Korea, between July 2024 and June 2025. The ROC curves illustrate the discriminative performance of 3 models for distinguishing CN individuals from those with MCI: the full 5-task model (red line, AUC=0.861), the primary 4-task model (blue line, AUC=0.787), and the demographic-adjusted model including age and education (green line, AUC=0.794). The comparison indicates comparable classification performance after adjustment for demographic covariates. AUC: area under the receiver operating characteristic curve; CN: cognitively normal; MCI: mild cognitive impairment; ROC: receiver operating characteristic.

To evaluate the potential influence of demographic covariates, age and education were added as input features in a sensitivity analysis (Table 5). The AUC showed a modest increase from 0.787 (95% CI 0.643‐0.932) to 0.794 (95% CI 0.654‐0.934; Figure 3). The balanced accuracy of the primary 4-task model was 80.1%, showing minimal difference from the overall accuracy (55/67, 82.1%), which suggests that the model maintains consistent classification performance across groups. In the demographic-adjusted model, the balanced accuracy was 77.9%. This slight numerical shift indicates that while task-related features remain the primary drivers of diagnostic performance, demographic factors appeared to contribute incrementally to the model’s classification. Other performance indices remained broadly comparable to those of the primary 4-task model, with a sensitivity of 75% (15/20) and specificity of 80.9% (38/47) in the demographic-adjusted model.

As shown in Figure 2, the overall pattern of variable importance appeared to remain similar after demographic adjustment. Specifically, the sale items task variables (X3_1 and X3_2) continued to rank among the top predictors in both the primary 4-task and demographic-adjusted models. Age also showed a relatively notable contribution, ranking third in the importance hierarchy, although its inclusion did not appear to substantially change the overall diagnostic pattern. Education, by contrast, had a relatively low importance ranking, placing near the bottom of the feature set (9th; mean decrease Gini=1.7883). Taken together, these findings suggest that the model’s classification performance was still mainly related to task performance, while the influence of demographic factors may have been limited (Table 6).

**Table 5.** Confusion matrix for the demographic-adjusted model. This cross-sectional diagnostic study involved community-dwelling older adults (N=67) recruited from the Yuseong Senior Welfare Center in Daejeon, South Korea, between July 2024 and June 2025.

	Observed CN^a^, n	Observed MCI^b^, n
Predicted CN	38	5
Predicted MCI	9	15

a CN: cognitively normal.

b MCI: mild cognitive impairment.

**Table 6.** Comparison of classification performance across the 3 predictive models. This cross-sectional diagnostic study involved community-dwelling older adults (N=67) recruited from the Yuseong Senior Welfare Center in Daejeon, South Korea, between July 2024 and June 2025. The table compares the diagnostic performance of the primary 4-task model (excluding the puzzle-solving task) and the demographic-adjusted model (including age and education) for distinguishing cognitively normal individuals from those with mild cognitive impairment. Classification performance was evaluated based on out-of-bag predictions from random forest models. Balanced accuracy is reported to account for potential group imbalance.

Model	Accuracy, n/N (%)	Balanced accuracy, %	Sensitivity, n/N (%)	Specificity, n/N (%)
Full 5-task	53/67 (79.1)	76.5	14/20 (70.0)	39/47 (83.0)
Primary 4-task model	55/67 (82.1)	80.1	15/20 (75.0)	40/47 (85.1)
Demographic-adjusted model	53/67 (79.1)	77.9	15/20 (75.0)	38/47 (80.9)

### Convergent Validity and Clinical Correlation

The convergent validity of the digital tool was assessed by calculating Pearson correlation coefficients between DCS1 and DCS2 and established clinical standards (CERAD-K2 total score and MMSE). The correlation analysis revealed that both DCS1 and DCS2 showed statistically significant, moderate relationships with the CERAD-K2 total score (DCS1: *r*=.655; DCS2: *r*=.447; both *P*<.001; Table 7). Similar trends were observed in comparisons with the MMSE, with DCS1 (*r*=.518, *P*<.001) showing a slightly stronger correlation than DCS2 (*r*=.442, *P*<.001). DCS1, which is based on accuracy scores, exhibited a slightly higher correlation with traditional clinical standards compared to DCS2, which integrates RT features. These moderate correlations are consistent with the ranges typically observed in digital screening tools and provide evidence for the convergent validity of the proposed scenario-based assessment.

**Table 7.** Pearson correlations between digital composite scores and clinical standards. Pearson correlations between digital composite scores and clinical reference measures in a cross-sectional diagnostic study involving community-dwelling older adults classified as cognitively normal status or as having mild cognitive impairment. Participants were recruited from the Yuseong Senior Welfare Center in Daejeon, South Korea, from July 2024 to June 2025.

Variable	CERAD-K2^a^ total score, r (95% CI)	*P* value	MMSE^b^, r (95% CI)	*P* value
DCS1^c^	0.655 (0.493-0.774)	<.001	0.518 (0.317-0.674)	<.001
DCS2	0.447 (0.232-0.621)	<.001	0.442 (0.226-0.617)	<.001

a CERAD-K2: Consortium to Establish a Registry for Alzheimer's Disease Assessment Packet, Korea, second edition.

b MMSE: Mini-Mental State Examination.

c DCS: digital composite score.

### Subjective Workload and Tool Feasibility

To assess the feasibility and user experience of the digital tool, perceived workload was evaluated using the NASA-TLX immediately following the task administration. Total workload scores did not differ between groups (*P*=.97), with similar mean ratings observed in the CN group (2.57, SD 0.71) and group with MCI (2.58, SD 0.58). These findings suggest that the scenario-based serious game was perceived as having a comparable cognitive demand across groups, indicating its feasibility for use among older adults in community settings.

## Discussion

### Principal Findings

This cross-sectional diagnostic accuracy study developed and evaluated a culturally adapted, tablet-based, scenario-based serious game for community-based MCI screening among Korean older adults, providing preliminary evidence of its feasibility and diagnostic utility. The final classification model demonstrated promising diagnostic performance, with an overall accuracy of 82.1% (55/67), a sensitivity of 75% (15/20), and a specificity of 85.1% (40/47). The robustness of the classification framework was further supported by a series of sensitivity analyses. Model performance remained relatively consistent despite variations in task composition and the inclusion of demographic covariates. In addition, the findings suggest the potential value of incorporating task-level RT as a process-sensitive behavioral indicator alongside conventional accuracy measures.

A notable finding emerged from the exploratory evaluation of the initial 5-task battery. Although the initial 5-task model achieved an accuracy of 79.1% (53/67), the puzzle-solving task was associated with a higher rate of false-negative classifications, particularly among participants with MCI. In a post hoc sensitivity analysis, excluding this task increased sensitivity from 70% (14/20) to 75% (15/20), while overall accuracy and specificity remained comparable. However, the AUC decreased from 0.861 to 0.787, indicating that improvements in threshold-dependent performance were accompanied by reduced overall discriminative ability. These findings underscore the potential value of task-level analysis in serious game design, suggesting that identifying tasks with limited discriminative value may inform future refinement of digital screening tools and strengthen process-based cognitive assessment.

Furthermore, we examined the potential confounding effects of demographic variables, a common concern in digital cognitive assessment [54,55]. In the demographic-adjusted model, the inclusion of age and education as input features was associated with an AUC of 0.794 and an overall accuracy of 79.1% (53/67). Feature importance analysis consistently identified both the accuracy and RT measures from the sale items task as the most important predictors across both models. These findings suggest that task-specific accuracy and RT measures, particularly those derived from the sale items task, contributed more to model performance than the demographic variables included in the model.

Finally, the assessment of convergent validity and feasibility provides additional support for the clinical and practical utility of this tool. DCS1 and DCS2 showed statistically significant, moderate correlations with established clinical measures, including the CERAD-K2 total score and the MMSE. Moreover, the lack of significant group differences in NASA-TLX workload scores suggests that the game-based format imposed a comparable subjective workload on CN and participants with MCI. Taken together, these findings suggest that this culturally adapted, serious game–based approach may provide a scalable and user-friendly platform for community-based MCI screening and cognitive monitoring.

### Interpretation of Accuracy and RT Markers

The diagnostic utility of the serious game–based tool may stem from its ability to capture both task accuracy and RT within cognitively meaningful tasks. Feature importance analysis identified accuracy (X3_1) in the sale items task as the strongest predictor, followed by the RT (X3_2) from the same task. These findings may have neuropsychological relevance, as the sale items task is designed to reflect visual-verbal episodic memory, a domain known to be affected early in amnestic MCI and the prodromal phase of Alzheimer disease [3,4].

The finding that both task-level accuracy and RT contributed to classification suggests that the tool captures not only successful task performance but also differences in processing efficiency associated with memory retrieval. In early-stage MCI, individuals may sometimes reach the correct answer but require more time and cognitive effort to do so [64]. By quantifying these subtle delays in responding within an ecologically relevant shopping scenario, the tool may help address some limitations of traditional accuracy-based assessments, which are susceptible to ceiling effects [18,38,39].

In contrast, the relatively low contribution of the puzzle-solving task in the initial 5-task model may offer insights into the cognitive profile of early-stage decline. This task primarily reflects visuoconstructional praxis, a domain that has been reported to remain relatively preserved during the early stages of MCI [65]. Consequently, the relatively preserved performance of some participants with MCI on this task, particularly those misclassified as CN, may have contributed to its limited discriminative power within the sample. These findings suggest that the tool may be particularly sensitive to episodic memory deficits, which represent one of the earliest cognitive changes observed in MCI [3,4,13]. More broadly, they indicate that refining the task battery based on the discriminative contribution of individual tasks may improve the diagnostic performance of serious game–based cognitive screening tools.

### Comparison With Prior Work

When compared with widely used cognitive screening instruments, the diagnostic performance of the serious game–based tool appears broadly comparable. Traditional screening tools such as the MMSE often show modest sensitivity for MCI (around 60%‐65%), partly due to ceiling effects and limited coverage of executive or attentional domains [18,66]. The MoCA and CERAD-K2 total scores generally yield sensitivities of approximately 80%‐85% [67,68] and 79%‐84% [69,70], respectively. However, both require trained examiners, with the more comprehensive CERAD-K2 requiring longer administrations [16,53], which may limit their scalability for routine community screening. Within this context, the performance of the tool (accuracy 55/67, 82.1%; sensitivity 15/20, 75%; and specificity 40/47, 85.1%) appears broadly similar to these established approaches while relying on automated, tablet-based task performance measures that may help reduce examiner-related variability. Notably, the automated tool achieved this level of diagnostic performance within approximately 20 minutes, supporting its feasibility for use in both clinical and community settings.

Beyond traditional instruments, the landscape of digital MCI screening is increasingly shifting toward ecologically grounded and culturally adapted approaches that prioritize real-world functional markers over abstract performance metrics [24,31,35-37]. In this expanding field, the present results broadly align with performance ranges reported in contemporary digital and serious game–based MCI screening studies [26,32,71-74]. To place these findings in the context of current domestic digital screening tools, it is useful to compare the present results with those of the recently developed Seoul Cognitive Status Test (SCST) [75]. As reported in a recent clinical utility study involving a large outpatient cohort [75], the SCST demonstrated robust diagnostic validity in clinical settings. Notably, while the SCST achieved near-ceiling discriminative performance for dementia (AUC 0.980), distinguishing clinically normal individuals from those with MCI remained more challenging (AUC 0.854). Although the study populations differed, the comparable performance observed in the present study suggests that ecologically grounded serious game–based assessment may achieve clinically meaningful diagnostic performance in community settings. Furthermore, the tool achieved a specificity of 85.1% (40/47), providing a balanced diagnostic profile suitable for community-level screening. Maintaining this balance is important, as it may help mitigate the risk of excessive false positives that could otherwise strain health care referral systems [76,77].

A key strength of the tool lies in its integrated, scenario-based narrative structure. Unlike many existing digital platforms consisting of isolated mini-games or simple computerized versions of abstract tasks [26,71], the tool integrates multidomain assessment within a seamless daily-life simulation (eg, market shopping and navigating traffic), while capturing both accuracy and task-level RT. Furthermore, the tool was designed to facilitate real-world implementation and scalability for community-based screening. The tool demonstrated comparable diagnostic performance using a standard tablet interface without the need for specialized hardware (eg, high-end VR sensors), which may limit the large-scale deployment of some contemporary high-tech systems [78,79].

Finally, a notable aspect of the tool is its culturally adapted design, developed specifically for community-dwelling Korean older adults. While many existing digital cognitive assessment tools rely on abstract or linguistically neutral tasks that may not fully reflect the sociocultural contexts of non-Western populations [35-37], the present serious game embeds cognitive tasks within familiar, culturally relevant scenarios, such as participating in traditional games. This approach may enhance ecological validity while potentially reducing cultural bias in the assessment process [31]. Such a design aligns with emerging global initiatives toward the indigenization of cognitive assessments, in which researchers are moving beyond literal translations to develop digital tools grounded in local sociocultural contexts [36,37,80]. Recent efforts, such as the CARE study [80], which developed game-based cognitive protocols for older adults in India, highlight the importance of incorporating indigenous perspectives. By integrating Korean-specific cognitive tasks, the tool reflects this broader movement toward culturally grounded digital assessment and may provide a useful framework for developing culturally adapted cognitive screening tools in other cultural settings.

### Clinical and Ecological Implications

The integration of RT variables across multiple tasks suggests that time-based measures may provide clinically relevant information that complements traditional accuracy-based scores. Slower RT has been recognized as a potential behavioral indicator of age-related changes in processing efficiency [81-83], although such information is not routinely captured by memory-centered, accuracy-focused assessments such as the CERAD-K2 [15,23]. Within the present tool, RT indicators embedded in ecologically structured scenarios may capture aspects of processing inefficiency that are not fully reflected by accuracy measures alone. Incorporating both accuracy and RT measures may, therefore, contribute to a more differentiated characterization of cognitive performance in older adults, potentially supporting a more process-oriented understanding of cognitive aging.

The scenario-based design of this tool may offer a practical alternative to traditional cognitive assessment batteries, particularly in community settings. Conventional tests such as the CERAD-K2 rely on abstract tasks (eg, word list recall) that are not explicitly embedded in everyday contexts. In contrast, the present tool assesses cognition through familiar activities such as recalling sale items at a market, navigating traffic, and route finding. This ecological approach may help capture functional aspects of cognition that are relevant to independent living [84,85]. Specifically, these tasks are designed to align with instrumental activities of daily living, which are often among the first functional domains to be affected in the early stages of cognitive impairment [86]. By quantifying cognitive performance within these functional contexts, the tool may provide indicators of early cognitive changes relevant to independent living. Moreover, presenting these tasks within a continuous daily-life narrative may enhance user engagement and acceptability, supporting the feasibility of community-based screening [87,88].

### Limitations

Despite the promising performance of the tool, several limitations should be acknowledged. First, although the sensitivity of 75% (15/20) is comparable to that of established clinical instruments, the corresponding false-negative rate of 25% (5/20) should be acknowledged. Therefore, the tool should be considered a complementary screening aid rather than a standalone diagnostic approach, particularly in community-based screening. Second, the generalizability of the findings is constrained by the sample characteristics. Participants were recruited through convenience sampling from a single center and demonstrated relatively higher educational attainment than the national average for Korean older adults [89], which may limit the generalizability of the findings to populations with different educational and sociodemographic backgrounds. Third, some participants required occasional assistance with navigation or device handling, suggesting that further interface refinement may be needed to support fully independent administration [90,91]. Nevertheless, the present version appeared feasible for supervised community-based administration. Fourth, although the RF classifier and out-of-bag procedure were used to estimate classification performance, the relatively small and imbalanced class distribution should be considered when interpreting the findings. Moreover, these performance estimates reflect internal rather than external validation. The post hoc exclusion of the puzzle-solving task was an exploratory refinement intended to identify potentially discriminative markers, and these findings require confirmation in an independent cohort. Finally, because this was a cross-sectional study, the present findings cannot address longitudinal predictive validity for dementia conversion or long-term cognitive trajectories [6,92].

### Future Directions

Future research should validate this tool in independent, multisite cohorts. Such studies are needed to confirm the robustness and generalizability of the current findings beyond the internal out-of-bag validation used in the present study. Longitudinal follow-up studies will also be needed to determine whether the RT-based and scenario-based indicators identified here are predictive of cognitive decline over time, including conversion from MCI to dementia. In addition, broader validation across individuals with diverse educational backgrounds and varying levels of digital literacy will be important for assessing real-world scalability [93,94]. Further studies should also incorporate additional variability-based behavioral markers, such as trial-to-trial response-time variability, intraindividual SD, and error-pattern metrics, to better capture response inconsistency and subtle inefficiencies associated with early cognitive decline [95,96]. Finally, continued refinement of autonomous usability features, along with further exploration of adaptive task parameters, may help improve sensitivity to subtle early cognitive changes.

### Conclusions

This study provides preliminary evidence supporting the diagnostic utility and feasibility of a tablet-based, scenario-based serious game for community-based MCI screening. The key innovation of this study lies in integrating culturally familiar, ecologically meaningful daily-life scenarios with task-specific accuracy and RT measures. This approach captures both task performance accuracy and potential differences in processing efficiency within a continuous narrative context. Unlike conventional accuracy-focused screening instruments and digital tools composed of isolated or abstract tasks, this approach combines culturally adapted task design with task-specific RT measures that may provide information about processing efficiency in addition to conventional accuracy measures. These findings contribute to the growing field of digital cognitive assessment by demonstrating the potential value of culturally adapted and ecologically grounded serious games for MCI screening. From a practical perspective, the automated, tablet-based format may offer a scalable and accessible screening option for community settings without requiring specialized equipment. Nevertheless, independent, multisite studies with longitudinal follow-up are needed to establish the generalizability and long-term predictive utility of the tool.

## Supplementary material

10.2196/92334 Multimedia Appendix 1 Screenshots of the serious game for mild cognitive impairment screening.

## Abbreviations

AUC: area under the receiver operating characteristic curve
CERAD-K2: Consortium to Establish a Registry for Alzheimer's Disease Assessment Packet, Korea, second edition
CN: cognitively normal
DCS: digital composite score
*DSM-5*: *Diagnostic and Statistical Manual of Mental Disorders* (Fifth Edition)
MCI: mild cognitive impairment
ML: machine learning
MMSE: Mini-Mental State Examination
MoCA: Montreal Cognitive Assessment
NASA-TLX: NASA Task Load Index
RF: random forest
RT: reaction time
SCST: Seoul Cognitive Status Test
