# Supplementary material for: Scenario-Based Serious Game for Screening Mild Cognitive Impairment in Older Adults: Cross-Sectional Preliminary Validation Study
Source: JMIR Serious Games. 2026 Sep 16;14:e92334. doi: 10.2196/92334 (PMC13581261; doi:10.2196/92334)
Supplement: Supplementary file 1 [file games-v14-e92334-s001.doc]

**Figure S1.** Sale item task.

**Figure S2.** Puzzle-solving.

**Figure S3.** Traffic signal.

**Figure S4.** Rice-barley go/no-go.

**Figure S5.** Spatial navigation.
